# IRE1-mTOR-PERK Axis Coordinates Autophagy and ER Stress-Apoptosis Induced by P2X7-Mediated Ca^2+^ Influx in Osteoarthritis

**DOI:** 10.3389/fcell.2021.695041

**Published:** 2021-06-17

**Authors:** Zihao Li, Ziyu Huang, He Zhang, Jinghan Lu, Yingliang Wei, Yue Yang, Lunhao Bai

**Affiliations:** ^1^Department of Orthopedics, Shengjing Hospital of China Medical University, Shenyang, China; ^2^Foreign Languages College, Shanghai Normal University, Shanghai, China

**Keywords:** P2X7 receptor, autophagy, endoplasmic reticulum stress, protein kinase R-like endoplasmic reticulum kinase, inositol-requiring enzyme-1, mammalian target of rapamycin, osteoarthritis, treadmill exercise

## Abstract

Moderate-intensity exercise can help delay the development of osteoarthritis (OA). Previous studies have shown that the purinergic receptor P2X ligand gated ion channel 7 (P2X7) is involved in OA development and progression. To investigate the effect of exercise on P2X7 activation and downstream signaling in OA, we used the anterior cruciate ligament transection (ACLT)-induced OA rat model and primary chondrocyte culture system. Our *in vivo* experiments confirmed that treadmill exercise increased P2X7 expression and that this effect was more pronounced at the later time points. Furthermore, P2X7 activation induced endoplasmic reticulum (ER) stress and increased the expression levels of ER stress markers, such as 78 kDa glucose-regulated protein (GRP78), protein kinase R-like endoplasmic reticulum kinase (PERK), inositol-requiring enzyme-1 (IRE1), and activating transcription factor 6 (ATF6). At the early time points, IRE1 and PERK were activated, and mTOR was inhibited. At the later time points, mTOR was activated, mediating PERK to promote ER stress-apoptosis, whereas IRE1 and autophagy were inhibited. To confirm our observations *in vitro*, we treated primary chondrocytes with the P2X7 agonist benzoylbenzoyl-ATP (Bz-ATP). Our results confirmed that P2X7-mediated Ca^2+^ influx activated IRE1-mediated autophagic flux and induced PERK-mediated ER stress-apoptosis. To further investigate the role of P2X7 in OA, we injected mTOR antagonist rapamycin or P2X7 antagonist A740003 into the knee joints of ACLT rats. Our results demonstrated that mTOR inhibition induced autophagy, decreased apoptosis, and reduced cartilage loss. However, injection of mTOR agonist MHY1485 or Bz-ATP had the opposite effect. In summary, our results indicated that during the early stages of moderate-intensity exercise, P2X7 was activated and autophagic flux was increased, delaying OA development. At the later stages, P2X7 became over-activated, and the number of apoptotic cells increased, promoting OA development. We propose that the IRE1-mTOR-PERK signaling axis was involved in the regulation of autophagy inhibition and the induction of apoptosis. Our findings provide novel insights into the positive and preventative effects of exercise on OA, suggesting that the intensity and duration of exercise play a critical role. We also demonstrated that on a molecular level, P2X7 and its downstream pathways could be potential therapeutic targets for OA.

## Introduction

Osteoarthritis (OA) is a joint degenerative disease that has a strong impact on the quality of life in elderly individuals, leading to reduced mobility, pain, and even disability. This disease also adds a heavy burden to health care systems (Hunter et al., 2014; Sharma, 2021). OA is characterized by the destruction of cartilage, including aberrant chondrocyte metabolism, and degradation of the extracellular matrix (Evans, 2018). We previously reported that moderate-intensity exercise effectively alleviated cartilage loss and OA symptoms (Zhang et al., 2019). We also found that purinergic receptor P2X ligand gated ion channel 7 (P2X7) was involved in OA incidence and development (Li et al., 2021b); furthermore, its expression levels were closely related to biomechanical stress (Li et al., 2021a). However, it is still not known whether moderate-intensity exercises affect OA via the regulation of P2X7 expression.

P2X7 belongs to the family of purinergic receptor proteins. As a cation channel gated by adenosine triphosphate (ATP), P2X7 mediates K^+^ efflux and Na^+^ and Ca^2+^ influx, resulting in various cellular responses, including the release of inflammatory cytokines and pyroptosis (Ferrari et al., 1997), as well as increased production of free radical and reactive oxygen species (ROS) (Cruz et al., 2007; Fontanils et al., 2010). Furthermore, biomechanical forces have been shown to regulate the expression of P2X7. It has been reported that P2X7 expression levels are differentially regulated by exercise. Sedentary individuals have the lowest expression, while individuals with high fitness levels have the highest expression; moreover, following exercise, P2X7 expression decreases (Comassi et al., 2017). On a molecular level, mechanical stimulation induces P2X7 activation via ATP (Grol et al., 2009), resulting in Ca^2+^ influx. Benzoylbenzoyl-ATP (Bz-ATP), a selective P2X7 agonist, has been reported to have a similar effect on osteoclasts, the bone-resorbing cells, indicating that this purinergic receptor plays a role in bone turnover (Naemsch et al., 2001); however, the activation status of P2X7, or its expression level, in response to exercise during OA has not been investigated yet.

Mechanical load can affect the metabolic balance of chondrocytes and reduce OA symptoms via the regulation of endoplasmic reticulum (ER) stress and autophagy (Zheng et al., 2019). Excessive mechanical stress increases Ca^2+^ concentration in the ER lumen, leading to Ca^2+^ overload, accumulation of misfolded proteins in the ER lumen, and induction of ER stress (Zhang and Kaufman, 2008; Liu et al., 2015). As a result, to promote cell survival, unfolded protein response (UPR) is initiated. To prevent further translational overload of the ER, three UPR receptor proteins are activated: protein kinase R-like endoplasmic reticulum kinase (PERK), inositol-requiring enzyme-1 (IRE1), and activating transcription factor 6 (ATF6) (Malhotra and Kaufman, 2007). To restore ER homeostasis, PERK and IRE1 delay mRNA translation and reduce the protein load in the ER, while IRE1 activation induces autophagy (Walter and Ron, 2011). In addition, ATF6 increases protein folding ability by dissociating from 78 kDa glucose-regulated protein (GRP78), the ER chaperone responsible for protein folding. During prolonged ER stress, instead of cell survival, the cleavage and activation of caspase-12 lead to cell apoptosis (Rao et al., 2004; Hetz, 2012). This process, also referred to as ER stress pathway-apoptosis (ER stress-apoptosis), is mainly mediated by PERK. PERK phosphorylation increases the expression of C/EBP homologous protein (CHOP) and caspase-12, as well as the activation of cleaved caspase-3 (Uehara et al., 2014). In addition to Ca^2+^ dysregulation, changes in the redox state also induce ER stress. ATP activates P2X7-mediated Ca^2+^ influx and stimulates ROS production (Bidula et al., 2019). ROS, in turn, induce oxidative stress, leading to mitochondrial damage and ER stress, triggering the apoptotic cascade. Since ROS have been reported to play a role in OA, it is possible that P2X7-mediated regulation of ER stress-apoptosis would affect chondrocytes, resulting in the progression of OA (Lepetsos and Papavassiliou, 2016).

As mentioned earlier, autophagy is one of the pathways activated by UPR. Autophagy is a tightly regulated process that allows cells to remove damaged organelles, abnormal proteins, and other macromolecules, first, by forming autophagosomes around the target, and then by fusing with lysosomes to generate autolysosomes (Klionsky et al., 2021), leading to the degradation of that cargo (Yang and Klionsky, 2010). There is a connection between autophagy and OA; during OA development, increased autophagic flux effectively alleviates the mitochondrial instability and ER stress caused by ROS via selective autophagy (Tang et al., 2017). Autophagy is a dynamic process, and two main markers of autophagy, Beclin-1 and microtubule-associated protein 1 light chain 3 beta (LC3B), are commonly used to characterize autophagic flux. LC3B is the major component of autophagosomal membranes, and conversion of the cytosolic LC3B-I into the membrane-associated lipidated LC3B-II indicates autophagosome formation, while elevated levels of both Beclin-1 and LC3B suggest the increased formation of autophagosomes. To characterize the formation of autolysosomes, colocalization of LC3B with lysosomal associated membrane protein 2 (LAMP2) is commonly used to confirm the fusion of autophagosomes and lysosomes.

The initiation of autophagy is tightly controlled by mammalian target of rapamycin (mTOR), a negative regulator of autophagy and a downstream target of P2X7. During the early stages of P2X7 activation, the Ca^2+^ influx induces mTOR signaling, leading to the inhibition of lysosome biogenesis and autophagy. During the later stages of P2X7 activation, the lysosomal stability is disrupted, and the fusion of autophagosome and lysosome is blocked, resulting in apoptosis (Sekar et al., 2018).

We hypothesized that P2X7-mediated activation of mTOR is involved in the regulation of autophagy and apoptosis initiation in chondrocytes during exercise. To test this hypothesis, we utilized a moderate-intensity treadmill exercise intervention using the rat anterior cruciate ligament transection (ACLT) OA model. Based on the changes in P2X7 expression levels, we assessed whether the exercise regimen influenced OA. We also examined the Ca^2+^ influx induced by P2X7 activation and its effects on IRE1-mediated autophagy and PERK-mediated ER stress-apoptosis in chondrocytes. Additionally, we examined the role of mTOR in OA-related autophagy and apoptosis.

## Materials and Methods

### Antibodies and Reagents

The antibodies used in this study were as follows: anti-P2X7 (Abcam, ab109054), anti-p-PERK (Cell Signaling Technology, 3192), anti-p-IRE1 (Abcam, ab48187), anti-GRP78 (Abcam, ab108613), anti-CHOP (Cell Signaling Technology, 2895), anti-caspase-12 (Abcam, ab62484), anti-p-mTOR (Abcam, ab109268), anti-LAMP2 (Abcam, ab125068), anti-LC3B (Abcam, ab192890), anti-Beclin-1 (Abcam, ab62557), anti-GAPDH (Proteintech, 10494-1-AP), and HRP-conjugated IgG (Beyotime, A0208). The reagents used in the experiments were as follows: P2X7 receptor agonist Bz-ATP (Sigma, B6396), P2X7 receptor antagonist A740003 (Sigma, A0862), bafilomycin A1 (Sigma, 196000), mTOR activator MHY1485 (Sigma, SML0810), mTOR inhibitor rapamycin (Sigma, V900930), PERK inhibitor GSK2606414 (Sigma, 516535), and IRE1 inhibitor 4μ8C (SML0949).

### Animals, Treadmill Exercise, and Knee-Joint Injections

Specific pathogen-free grade, 5-week-old male Sprague-Dawley (SD) rats (*n* = 90; 220–230 g) were purchased from HFK Bioscience (Beijing, China). Rats were acclimatized for 1 week under a sterile environment at suitable temperature and humidity with adequate food and water and 12 h of light/dark cycles. All animal experiments complied with the Animal Ethics Regulations of China Medical University (no. 2017PS237K). A rat-specific treadmill (ZH-PT, Zhongshidichuang Science & Technology Development, Co., Ltd., Beijing, China) was used to perform adaptive treadmill training (1 h, speed 8 m/min) of rats for 1 week (Monday to Friday, rest on Saturday and Sunday). The formal treadmill training (1 h; speed 12 m/min; Monday to Friday, with rest on Saturday and Sunday) and the knee injection were performed as previously described (Zhang et al., 2019). The OA model was developed via ACLT as previously described (Stoop et al., 2001). Rats were anesthetized in a sterile environment using phenobarbital. The rats (*n* = 90) were randomly divided into two groups: (1) sham + treadmill exercise and (2) ACLT + treadmill exercise. Next, the ACLT group was further divided into five injection groups, A740003, rapamycin, rapamycin + Bz-ATP, MHY1485, and MHY1485 + A740003. Each knee joint was injected with 50 μL of solution twice a week, and the control groups were injected with the same volume of saline, as previously described (Li et al., 2021b). The injection concentrations of A740003, Bz-ATP, rapamycin, and MHY1485 were the same as those used in our *in vitro* experiments. The detailed experimental layout is depicted in the Supplementary Figure 1. At four time points (2, 4, 6, and 8 weeks), the rats were euthanized with a phenobarbital overdose and samples were collected. Following sampling, a part of the knee joint tissue was examined by microcomputed tomography (micro-CT), and the rest was fixed and decalcified for subsequent experiments.

### Histological Analysis and Immunohistochemistry

The excess muscle and soft tissues from the knee joints were removed. Knee joints were fixed in 4% paraformaldehyde (Biosharp, BL539A) at 37°C for 48 h and decalcified in 10% EDTA-2Na (Biofroxx, 6381-92-6) at 37°C for 6 weeks. The decalcification solution was changed twice a week. Following dehydration, wax immersion, and paraffin embedding, tissues were sectioned with a microtome (MICROM, HM 340E, Thermo Fisher Scientific). Hematoxylin and eosin (H&E) staining was performed using an automatic staining machine (Leica) (machine program 2). After sections were deparaffinized (program 1), they were stained with Toluidine blue O (TB) cartilage staining solution (Solarbio; Cat. No. G2543) for 30 min. To perform immunohistochemistry (IHC), the sections were washed with phosphate buffered saline (PBS) and a universal two-step detection kit (ZSGB-BIO; Cat. No. PV-9000) was used. Antigen retrieval was performed using the IHC Enzyme Antigen Retrieval Reagent (Boster; Cat. No. AR0026). After the peroxidase was quenched, sections were blocked with serum, incubated with the primary antibody (1:100) overnight at 4°C, then with the secondary antibody (1:200) at 37°C for 1 h, and developed using diaminobenzidine. Finally, sections were counterstained with hematoxylin. To evaluate apoptosis, a terminal deoxynucleotidyl transferase dUTP nick-end labeling (TUNEL) staining kit (Abbkine, KTA2011) was used. Antigen retrieval was performed following the manufacturer’s instructions. Sections were permeabilized with 0.1% Triton (Solarbio) for 15 min and incubated with the reaction mixture overnight at 4°C, and the nuclei were counterstained with 4′,6-diamidino-2-phenylindole (DAPI). The stained sections were dehydrated in xylene, mounted using the neutral resin, and finally observed under a fluorescence microscope (Nikon; Cat. No. E800).

### Primary Chondrocyte Cultures

Sprague-Dawley rats (4-week-old, male, SPF grade) were euthanized in a sterile environment with an overdose of phenobarbital. The knee joint and femoral head from both lower limbs were dissected out; the cartilage was removed, and then cut into pieces. The cartilage fragments were washed with PBS, incubated in digestive solution I [protease K (30 min, 4 mg/mL; Roche)]: 5% culture medium = 1:6) at 37°C for 2 h, then incubated in digestive solution II (collagenase D (2 h, 1.6 mg/mL; Roche): 5% culture medium = 1:6) at 37°C for 4 h, and the cells were extracted every 30 min. Cells were plated in a culture flask at a density of 2,000 cells/mL; cultured at 37°C, 5% CO_2_, and 95% humidity for 7 days; and then subcultured. The cells were cultured in Dulbecco’s modified Eagle’s medium/F12 medium (Gibco, Thermo Fisher Scientific) containing 10% fetal bovine serum and 1% penicillin/streptomycin, and the medium was replaced every 3 days. Cells from the first or second passages were used for subsequent experiments.

### Cell Viability Assays and Intracellular Calcium Measurements

The primary chondrocytes (first passage) were cultured to a confluence of 90%, detached using trypsin (Sigma Aldrich), re-plated in a 96-well plate at a density of 5,000 cells/mL, and then cultured for another 2 days. The cells (second passage) were treated with Bz-ATP when they reached 80% confluence. At the end of experiments, cells were washed with PBS and incubated with 10 μL of the Cell Counting Kit (CCK)-8 detection solution (Beyotime; Cat. No. C0042) + 90 μL culture medium for 2 h at 37°C in the dark. Absorbance was detected at a wavelength of 450 nm using a microplate reader (Synergy H1; BioTek, United States), and corresponding cell viability values were calculated.

To measure the intracellular Ca^2+^ levels, cells were cultured as described above. At the end of the experiment, cells were washed with PBS and then incubated with Fluo-4 AM (Beyotime, S1060) working solution (1:500 dilution in PBS) for 30 min at 37°C in the dark. The above steps were repeated to ensure that the Fluo-4 AM was completely converted into Fluo-4 inside the cells. The fluorescence intensity of each group was detected using a microplate reader (excitation wavelength = 488 nm, emission wavelength = 512–520 nm).

### Caspase-3 Activity Assays

Second passage cells were treated with inhibitors when they reached 80% confluence. At the end of the experiment, cell supernatants were collected for later use. The adherent cells were detached using trypsin. The cells were centrifuged (600 × *g*, 4°C, 5 min), washed with PBS, and then lysed with 100 μL of lysis buffer (Beyotime, C1115). The lysates were pelleted (4°C, 16,000–20,000 × *g*, 10–15 min) and resuspended (0°C, 15 min). To determine caspase-3 enzyme activity, 50 μL of supernatants were incubated with the caspase-3 substrate solution [40 μL detection buffer + 10 μL Ac-DEVD-pNA (2 mM)].

### Flow Cytometry

Second passage cells at 80% confluence were treated with inhibitors. At the end of experiment, cells were washed with PBS, detached using trypsin, centrifuged at 800 × *g*, at 4°C, for 5 min, washed again with PBS, and then resuspended in a working solution [1:50, Annexin-V: FITC Apoptosis Detection Kit (BD Biosciences, 556547)] to obtain the reaction solution. Annexin-V (5 μL) and propidium iodide (PI, 5 μL) were added to 100 μL of the reaction solution, mixed well, and then incubated at 37°C for 15 min in the dark. To detect ROS, cells were cultured as described and then incubated with 2′,7-dichlorodihydrofluor-escein diacetate (DCFH-DA) (1:1000, Beyotime, S0033S) at 37°C for 20 min. Subsequently, the cells were washed with a serum-free medium and then collected by trypsinization. Cells were analyzed by flow cytometry using an FACSCalibur instrument (BD Biosciences, United States). The Flow Jo software (BD Biosciences, version 10) was used for data analysis.

### Western Blotting

Second passage cells at 80% confluence were treated with inhibitors. At the end of experiment, cells were washed with PBS and lysed with radioimmunoprecipitation assay (RIPA) lysis buffer (Beyotime, P0013B). A cell scraper was used to collect the lysed mixture, and the lysates were incubated at 4°C for 30 min. After centrifugation (14,000 × *g*, 4°C, 20 min), supernatants were collected. A bicinchoninic acid protein assay kit (Beyotime; Cat. No. P0010) was used to measure the protein concentration. To denature the proteins, 5 × loading buffer was added, samples were boiled at 100°C for 10 min, and then stored at −20°C. To perform western blotting experiments, we used the PAGE Gel Fast Preparation Kit (Epizyme; Cat. No. PG112) and followed the manufacturer’s instructions for gel preparation, sample loading, electrophoresis (80 V, 30 min + 120 V, 60 min), membrane transfer (200 mA, 50/80 min), blocking (5% skim milk, 37°C, 2 h), primary antibody incubation (1:1000, 4°C, overnight), secondary antibody incubation (1:5000, 37°C, 2 h), and electrochemiluminescence (ECL) (GE, Thermo Fisher Scientific). The bands were statistically analyzed using the Image J software (NIH, United States) for gray values. GAPDH was used as an internal control.

### RNA Extraction and Quantitative Real-Time PCR Analysis

Second passage cells were treated with inhibitors until they reached 80% confluence. At the end of experiments, cells were washed with PBS and treated with TRIzol (TaKaRa; Cat. No. 9109), and RNA was extracted according to the manufacturer’s protocol. The NanoVne instrument (GE Healthcare) was used to measure the purity. Samples with A260/280 values between 1.8 and 2.0 were used for subsequent experiments. The Prime Script RT Master Mix (TaKaRa; Cat. No. RR036A) was used for reverse transcription, and TB Green Premix Ex Tag II (TaKaRa; Cat. No. RR820A) was used for amplification on a 7500 Real-Time PCR System (Applied Biosystems, Foster City, CA, United States). Thermocycling conditions were as follows: 95°C for 30 s, followed by 40 cycles at 95°C for 5 s and 60°C for 35 s. The obtained Ct values were normalized using the 2^–ΔΔCt^ formula, with *GAPDH* as the housekeeping gene. The primer sequences are listed in the Supplementary Figure 2.

### Immunofluorescence and Imaging Analysis

Primary chondrocytes (first passage) were plated in a fluorescent chamber (Biologix, 07-2108), treated with inhibitors, and cultured until they reached 50% confluence. At the end of the experiment, cells were washed with PBS, fixed in 4% paraformaldehyde at 37°C for 10 min, permeabilized in 0.1% Triton at 37°C for 10 min, and blocked with goat serum at 37°C for 30 min. Next, cells were incubated with the primary antibody (1:100, 4°C, overnight), and then with the fluorescent secondary antibody (1:200, 37°C, 1 h, in the dark), and the nuclei were counterstained with DAPI. Zeiss LSM880 confocal microscope (Zeiss, Oberkochen, Germany) was used for image acquisition. Image J software was used for image and statistical analyses.

### Transmission Electron Microscopy

First passage cells were cultured until cells reached 80% confluence and treated with inhibitors. At the end of experiment, cells were fixed with glutaraldehyde (2.5%, 37°C, 5 min, dark), collected with a cell scraper, and then centrifuged (1,000 × *g*, 37°C, 2 min). Next, cells were fixed with 1% OsO_4_ at 37°C for 30 min in the dark and resuspended. After dehydration, the cell pellet was embedded in resin. Ultrathin tissue sections (60 nm) were stained with uranyl acetate and lead citrate, and cell morphology and subcellular structures were then observed using transmission electron microscopy (TEM; Hitachi 800; Hitachi, Tokyo, Japan).

### Microcomputed Tomography

Collected knee joint tissues were fixed with 4% paraformaldehyde for 24–48 h, rinsed once with 75% alcohol, and then stored in 75% alcohol at 4°C, or tested immediately. A Skyscan 1276 (Bruker, Kontich, Belgium) and NRecon version 1.6 were used to analyze the proximal tibia and the subchondral bone area. Bone volume (BV), BV/total tissue volume (BV/TV), trabecular number (Tb.N), trabecular thickness (Tb.Th), and trabecular separation (Tb.Sp) were determined. CTAn version 1.9 was used for data analysis.

### Statistical Analysis

All experiments were performed at least three times. Data are expressed as means ± standard deviations. The between-group differences were determined using Student’s *t*-test or one-way analysis of variance with Tukey’s *post hoc* test in GraphPad Prism version 7.0c. Significance was set at *p* < 0.05.

## Results

### Treadmill Exercise Increases P2X7 Expression, Activates Early Stage Autophagy, and Induces ER Stress-Mediated Apoptosis During OA

**FIGURE 1:**
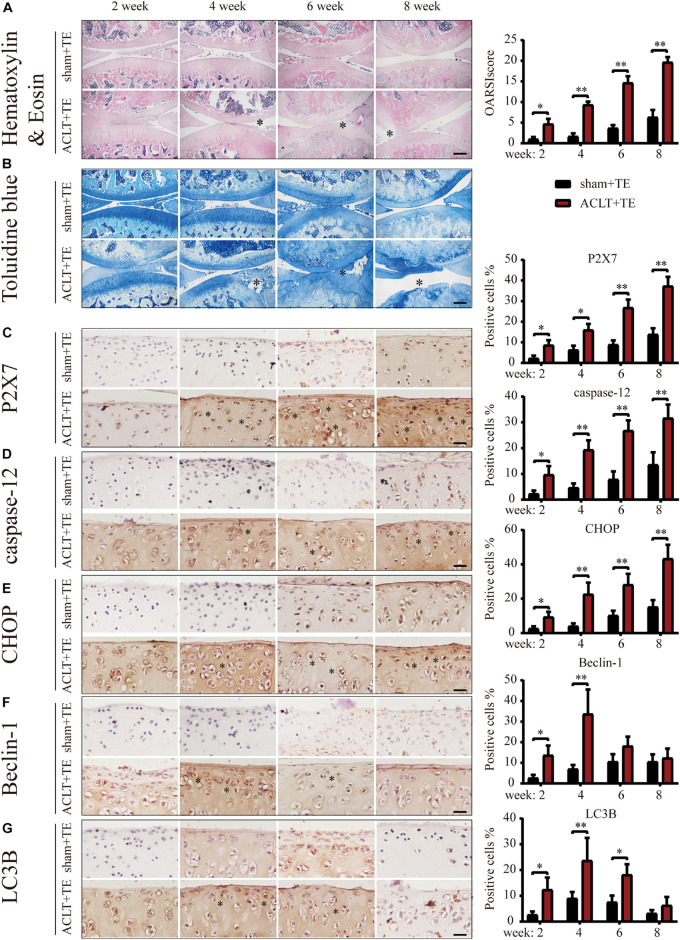
Effect of treadmill exercise on P2X7, autophagy, and apoptosis markers. H&E **(A)** and TB **(B)** staining of the sagittal sections of the knee joint tissue. The degree of cartilage loss and matrix degradation from 2–8 weeks (scale bar: 500 μm) was calculated using the Osteoarthritis Research Society International (OARSI) scoring system (see quantification on the right). **(C–G)** IHC staining and quantification. Expression level of **(C)** P2X7, the apoptosis markers caspase-12 **(D)** and CHOP **(E)**, and the autophagy markers LC3B **(F)** and Beclin-1 **(G)** over time (scale bar: 200 μm). Quantification of the stained cells is presented on the right. The asterisk in the HE and TB staining images represents the cartilage damage site, and the asterisk in the histochemical images represents the positively stained cells. Data are expressed as means ± standard deviations of at least three independent experiments. ^∗^*p* < 0.05, ^∗∗^*p* < 0.01.

We previously reported (Zhang et al., 2019; Li et al., 2021b) that P2X7 expression was increased in the rat OA model. Furthermore, we observed that treadmill exercises effectively alleviated OA occurrence and development. To investigate whether moderate intensity exercise can regulate the expression levels of P2X7, both the sham (control group) and ACLT (OA model group) rats performed treadmill exercises, and articular cartilage was evaluated at four different time points (2, 4, 6, and 8 weeks). The results of H&E and TB (Figures 1A,B) staining showed that rats from the ACLT group had higher levels of knee cartilage loss and matrix degradation, compared to the sham group at each time point. Furthermore, the severity of OA in the ACLT group increased with time as evaluated by the Osteoarthritis Research Society International (OARSI) score. IHC analysis showed (Figure 1C) that in the sham group, the expression levels of P2X7 increased only after 8 weeks of treadmill exercise; however, in the ACLT group, the expression level of P2X7 showed a difference in the second week, significantly increased by 6 weeks (between the sham group and the ACLT group), and gradually increased with time. The expression levels of the apoptosis indicators caspase-12 and CHOP (Figures 1D,E) also showed differences between the two groups within 2 weeks and significantly increased by 6 weeks. The expression of the autophagy indicators LC3B and Beclin-1 (Figures 1F,G) gradually increased between the second and fourth week and began to decrease by the sixth week. By 8 weeks, there were no significant differences between the groups.

**FIGURE 2:**
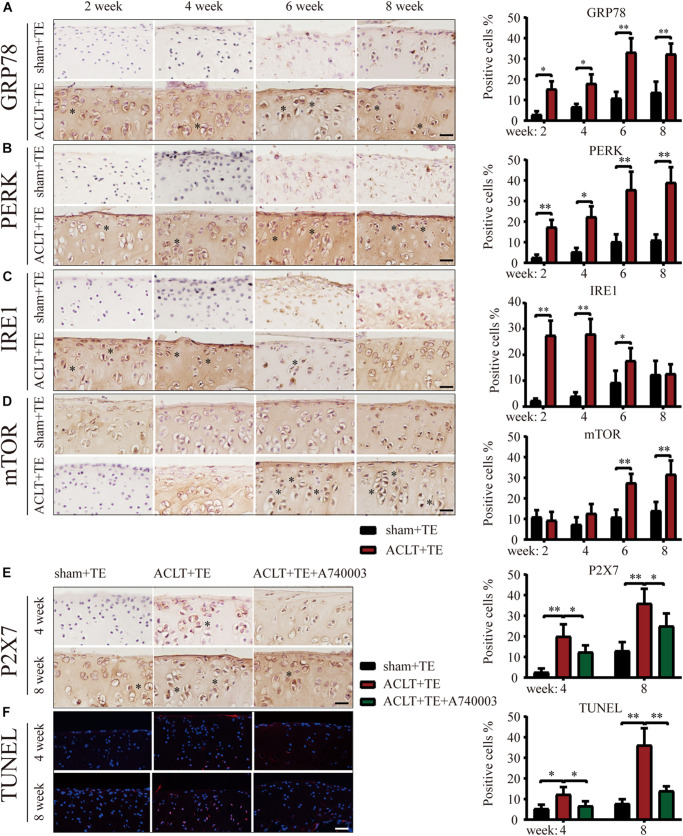
Treadmill exercise (TE) affects autophagy and ER stress-apoptosis through the regulation of P2X7 activity. IHC staining and quantification of the ER stress markers GRP78 **(A)**, PERK **(B)**, IRE1 **(C)**, and mTOR **(D)** were performed to observe the changes in their expression levels over time. Quantification of the stained cells is shown on the right. IHC staining and quantification of P2X7 expression levels **(E)** in the sham + TE, ACLT + TE, and ACLT + TE + A740003 groups at 4 and 8 weeks (scale bar: 200 μm). Quantification of the stained cells is presented on the right. TUNEL staining **(F)** showing the changes in the percentage of apoptotic chondrocytes following the intra-articular injection of the P2X7 inhibitor A740003 (scale bar: 200 μm). Quantification of the stained cells is presented on the right. The asterisk in the HE and TB staining images represents the cartilage damage site, and the asterisk in the histochemical images represents the positively stained cells. Data are expressed as means ± standard deviations of at least three independent experiments. ^∗^*p* < 0.05, ^∗∗^*p* < 0.01.

Next, we evaluated the effect of treadmill exercise on the induction of ER stress-apoptosis in OA. The results of IHC analysis demonstrated that the expression levels of the ER stress indicators GRP78, PERK, and IRE1 (Figures 2A–C) were significantly increased in the ACLT group compared to the sham group at all-time points, with the exception of IRE1 levels at week 8. Furthermore, the levels of GRP78 and PERK in the ACLT group increased with time, while the levels of IRE1 increased at the week 2 and 4 time points and then decreased down to the control level by week 8. Interestingly, the expression levels of mTOR (Figure 2D) were not significantly affected at the week 2 and 4 time points; however, mTOR levels were upregulated at the week 6 and 8 time points compared to the control group. Since the levels of autophagy markers Beclin-1 and LC3B were elevated in the ACLT group at the earlier time points (week 2 and 4) in response to treadmill exercise, these results suggested that autophagy was activated in the early stage of OA development and inhibited during the later stage. Moreover, mTOR expression levels were lower in the early stage but increased during the later time points. Since mTOR activation inhibits autophagy, our results suggest that mTOR may be involved in the regulatory transition between autophagy and apoptosis.

To explore whether the effect of treadmill exercise on the occurrence and development of OA was dependent on the expression levels of P2X7, rats in the ACLT group were injected the P2X7 receptor antagonist A740003, and samples were collected at 4 and 8 weeks. IHC analysis (Figure 2E) confirmed that the intra-articular injection of A740003 significantly reduced the expression of P2X7. Furthermore, our results showed that the inhibition of P2X7 effectively reduced the number of apoptotic chondrocytes as demonstrated by TUNEL staining (Figure 2F and Supplementary Figure 3). These findings indicate that treadmill exercise affects autophagy and apoptosis in chondrocytes through changes in P2X7 expression levels.

### Moderate and Excessive P2X7 Activation Has Opposing Effects on mTOR and Autophagy, and Both Induce ER Stress

**FIGURE 3:**
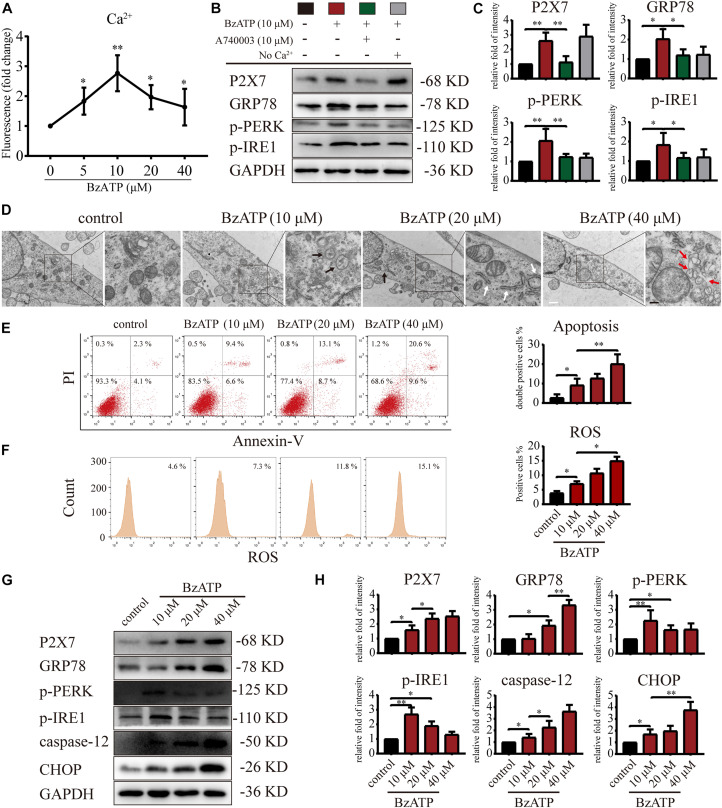
P2X7 activation triggers Ca^2+^ overload and induces ER stress. **(A)** Cells were cultured with different concentrations of Bz-ATP (0, 5, 10, 20, and 40 μM), the fluorescence signal was detected by the microplate reader, and the Ca^2+^ content in the ER was determined. **(B,C)** Cells were cultured with 10 μM Bz-ATP, 10 μM A740003, or Ca^2+^-free medium as described in section “Materials and Methods.” Western blotting was used to detect the protein levels of P2X7, GRP78, PERK, and IRE1. **(C)** Quantification of western blotting results. **(D)** Cells were cultured with different concentrations of Bz-ATP (0, 10, 20, and 40 μM) as described in section “Materials and Methods,” and TEM was used to evaluate cell morphology and subcellular structures (black arrows: autolysosomes; white arrows: the expansion of ER; red arrows: vesicles; white bar: 2 μm. black bar: 1 μm). **(E–F)** To detect the proportion of apoptotic cells and the level of oxidative stress, cells were stained with Annexin-V/PI and ROS and analyzed by flow cytometry. **(G,H)** Cells were cultured with different concentrations of Bz-ATP (0, 10, 20, and 40 μM), and the protein levels of P2X7, GRP78, p-PERK, p-IRE1, caspase-12, and CHOP were detected by western blotting. **(H)** Quantification of western blotting results. Data are expressed as means ± standard deviations of at least three independent experiments. ^∗^*p* < 0.05, ^∗∗^*p* < 0.01.

It has been previously reported that the selective activator Bz-ATP increases P2X7 expression and induces the OA phenotype in primary chondrocytes (Li et al., 2021b). The results of animal experiments (Figures 1, 2) showed that changes in the expression level of P2X7 regulate autophagy and apoptosis in chondrocytes. Therefore, we used Bz-ATP to investigate the connection between the different activation levels of P2X7 and autophagy and ER stress-apoptosis in primary chondrocytes *in vitro*. First, we incubated cells with different Bz-ATP concentrations (0, 10, 20, and 40 μM) for 6 and 12 h. The CCK-8 assay results demonstrated that cell viability was significantly affected at 12-h time point (Supplementary Figure 4A), therefore, 6-h time point was selected for all subsequent experiments. Next, we evaluated the effect of Bz-ATP on Ca^2+^influx (Figure 3A). Our results demonstrated that the change in fluorescence signal intensity was the largest at 10 μM, and the influx of Ca^2+^ gradually decreased as the Bz-ATP concentration increased. To evaluate the effect of Bz-ATP on ER stress, cells were incubated with P2X7 selective inhibitor A740003 in a Ca^2+^-free medium. Our results demonstrated that 10 μM Bz-ATP increased the protein levels of GRP78, PERK, and IRE1 (Figures 3B,C); however, protein levels decreased in response to P2X7 inhibitor or Ca^2+^ removal. The mRNA expression levels of these genes were affected in a similar manner (Supplementary Figure 4B).

**FIGURE 4:**
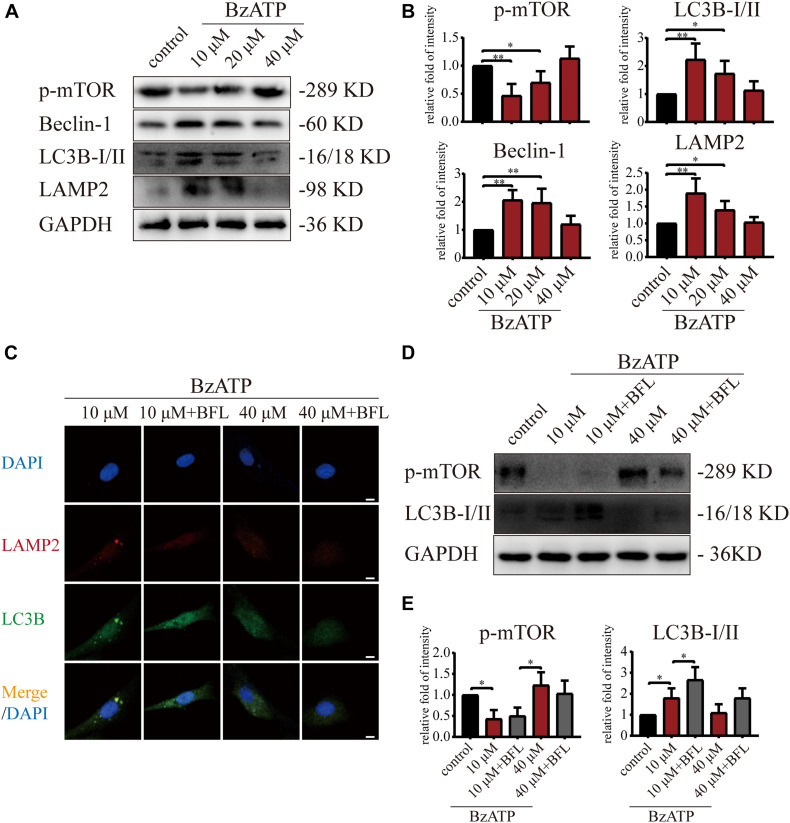
Moderate activation of P2X7 inhibits mTOR and activates autophagic flux. **(A,B)** Cells were cultured with different concentrations of Bz-ATP (0, 10, 20, and 40 μM), and the protein levels of p-mTOR, LC3B, Beclin-1, and LAMP2 were detected by western blotting. **(B)** Quantification of western blotting results. **(C)** Cells were cultured with 10 and 40 μM of Bz-ATP, with or without Bafilomycin A1 (BFL), and the localization of autophagosomes and lysosomes was evaluated using immunofluorescence (LC3B: green, LAMP2: red, DAPI: blue) (scale bar: 5 μm). **(D,E)** Cells were cultured with 10 and 40 μM of Bz-ATP, with or without Bafilomycin A1 (BFL), and western blotting was used to detect the protein levels of p-mTOR and LC3B. **(E)** Quantification of western blotting results. Data are expressed as means ± standard deviations of at least three independent experiments. ^∗^*p* < 0.05, ^∗∗^*p* < 0.01.

Next, we investigated the effect of P2X7 activation on autophagy and apoptosis in primary chondrocytes. TEM results showed that the cells in the control group had an intact nuclear membrane, tightly arranged ER, and no vesicles in the cytoplasm. As the concentration of Bz-ATP increased (10, 20, and 40 μM), the ER expanded, mitochondria swelled, and vesicles appeared in the cytoplasm. Furthermore, autophagosome and autolysosome-containing organelles were observed in the 10 μM group (Figure 3D). To investigate the effect of P2X7 activation on chondrocyte apoptosis and intracellular oxidative stress, cells were analyzed by flow cytometry. Our results showed that the percentage of apoptotic cells (Figure 3E) and ROS (Figure 3F) increased in response to higher Bz-ATP concentrations. Since redox and ion concentration disbalance are known to cause ER stress, we evaluated the expression of ER stress markers. Western blotting and qRT-PCR (Supplementary Figure 4C) showed that the expression levels of *P2X7*, *GRP78*, *caspase-12*, and *CHOP* (Figures 3G,H) gradually increased in response to P2X7 activation. Furthermore, the protein levels of p-PERK, p-IRE1 (Figures 3G,H), LC3B, Beclin-1, and LAMP2 (Figures 4A,B) were the highest at 10 μM Bz-ATP concentration and then gradually decreased. The difference was that the phosphorylation level of mTOR was inhibited by 10 μM Bz-ATP and then gradually increased.

We added Bafilomycin A1 (BFL) based on Bz-ATP intervention and set groups (control, Bz-ATP (10 μM), Bz-ATP + BFL, Bz-ATP (40 μM), and Bz-ATP + BFL). Immunofluorescence experiments (Figure 4C) showed that 10 μM Bz-ATP (but not 40 μM) increased the colocalization of LC3B and LAMP2. Furthermore, Bafilomycin A1 prevented the fusion of autophagosomes and lysosomes and increased the protein levels of LC3B without affecting p-mTOR (Figures 4D,E). Collectively, these results indicated that activation of P2X7 mediated Ca^2+^ influx, induced ER stress-apoptosis, and activated autophagy; however, the high levels of P2X7 activation inhibited autophagic flux, possibly via the inhibition of mTOR phosphorylation.

### mTOR Inhibits Autophagic Flux and Activates PERK to Induce ER Stress-Apoptosis

**FIGURE 5:**
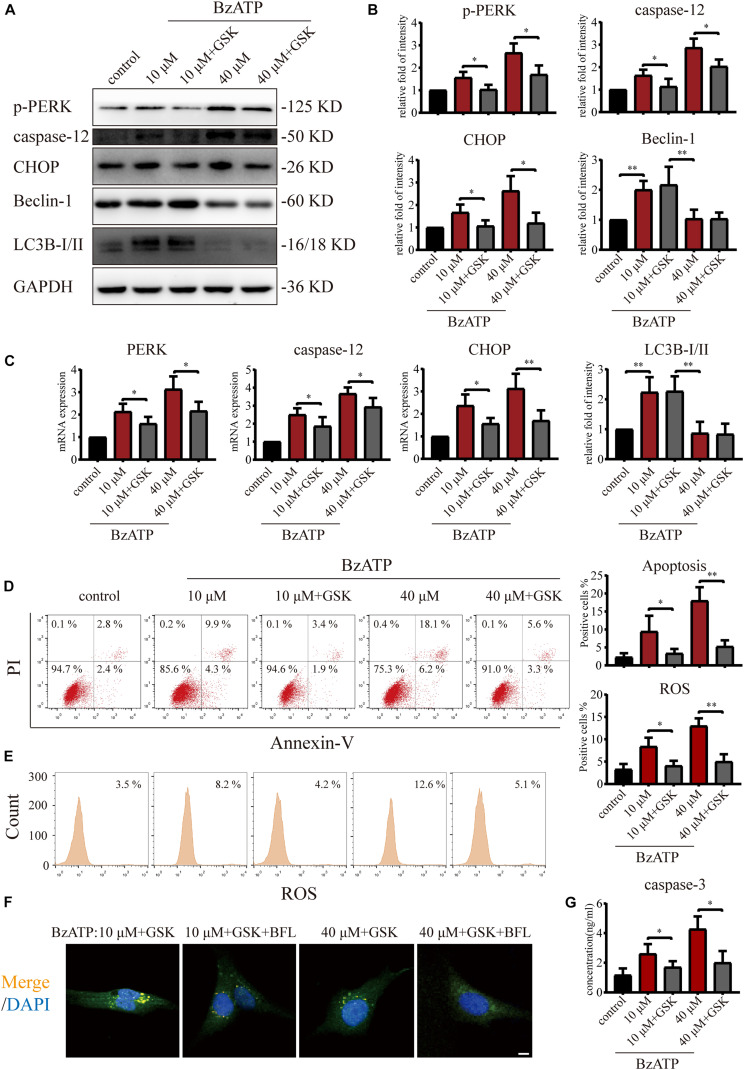
Effect of PERK inhibition on autophagic flux and ER stress-apoptosis. Cells were cultured with 10 and 40 μM of Bz-ATP, with or without 1 μM GSK2606414 (GSK). Western blotting **(A,B)** and qRT-PCR **(C)** were used to detect the protein and mRNA expression levels of *PERK*, *caspase-12*, *CHOP*, LC3B, and Beclin-1. Flow cytometry was used to detect the proportion of Annexin-V/PI double-stained cells **(D)** and the level of intracellular oxidative stress ROS **(E)**. **(F)** Cells were cultured with 10 μM Bz-ATP, 1 μM GSK2606414 (GSK), with or without Bafilomycin A1 (BFL). Immunofluorescence staining was used to observe the localization of autophagosomes and lysosomes (LC3B; green, Merge: yellow, DAPI: blue) (scale bar: 5 μm). **(G)** The caspase-3 activity assay; the statistical results are homogenized. Data are expressed as means ± standard deviations of at least three independent experiments. ^∗^*p* < 0.05, ^∗∗^*p* < 0.01.

Since PERK is located downstream of mTOR, to examine the role of PERK in the regulation of autophagy and apoptosis in response to P2X7, we cultured primary chondrocytes with PERK inhibitor GSK2606414 (GSK) using the following experimental design: control, Bz-ATP (10 μM), Bz-ATP + GSK, Bz-ATP (40 μM), and Bz-ATP + GSK. Western blotting (Figures 5A,B) and qRT-PCR (Figure 5C) results showed that GSK2606414 effectively decreased the protein and mRNA expression levels of *PERK*, *caspase-12*, and *CHOP*; however, it did not affect LC3B and Beclin-1. Annexin-V/PI (Figure 5D) and ROS (Figure 5E) were evaluated by flow cytometry and also confirmed the above conclusions. However, the percentage of apoptotic chondrocytes and the level of intracellular oxidative stress decreased significantly in response to GSK2606414. The results of the caspase-3 activity assay (Figure 5G) showed that GSK2606414 effectively alleviated cell apoptosis by inhibiting PERK. To further explore whether PERK affects the fusion of autophagosomes and lysosomes, we used the following experimental design: Bz-ATP (10 μM) + GSK, Bz-ATP + GSK + BFL, Bz-ATP (40 μM) + GSK, and Bz-ATP + GSK + BFL. Immunofluorescence experiments (Figure 5F and Supplementary Figure 5) demonstrated that GSK2606414 promoted the colocalization of LC3B and LAMP2, while BFL inhibited this colocalization. These results indicate that PERK prevented the fusion of autophagosomes and lysosomes, thus inhibiting autophagic flux and inducing ER stress-apoptosis.

**FIGURE 6:**
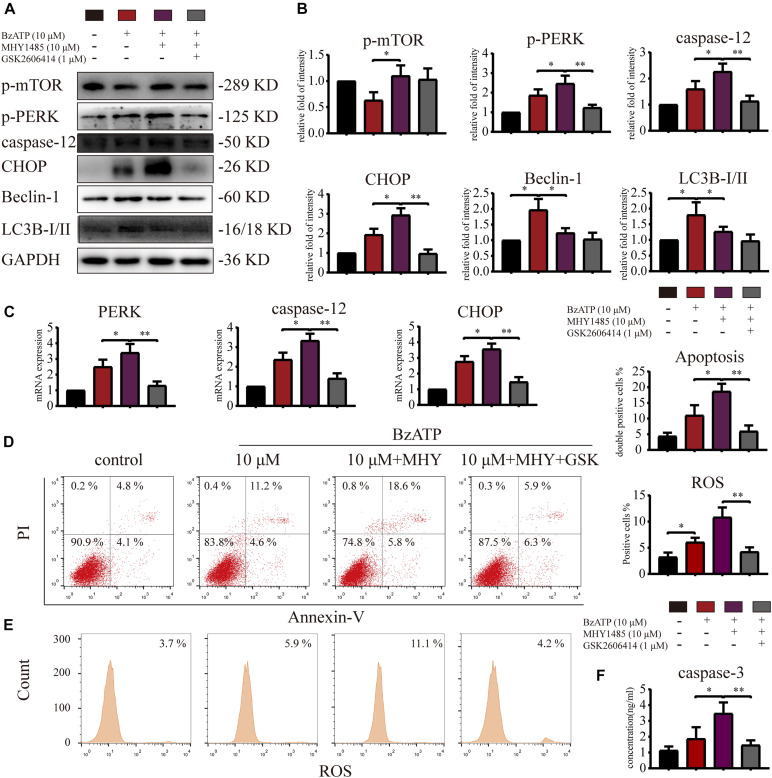
mTOR inhibits autophagy and promotes ER stress-apoptosis through PERK. Cells were cultured with 10 μM Bz-ATP, 1 μM GSK2606414 (GSK), with or without 10 μM MHY1485 (MHY). Western blotting **(A,B)** and qRT-PCR **(C)** were used to detect the protein and mRNA expression levels of mTOR, *PERK*, *caspase-12*, *CHOP*, LC3B, and Beclin-1. Flow cytometry was used to detect the percentage of Annexin-V/PI double-stained cells **(D)** and the level of intracellular oxidative stress ROS **(E)**. **(F)** The caspase-3 activity assay; the statistical results are homogenized. Data are expressed as means ± standard deviations of at least three independent experiments. ^∗^*p* < 0.05, ^∗∗^*p* < 0.01.

To investigate whether mTOR was involved in the regulation of autophagy and apoptosis in response to P2X7 stimulation, we treated chondrocytes with mTOR activator MHY1485 using the following experimental design: control, Bz-ATP (10 μM), Bz-ATP + MHY1485 (10 μM), and Bz-ATP + MHY1485 + GSK. Western blotting (Figures 6A,B) and qRT-PCR (Figure 6C) results demonstrated that MHY1485 increased the levels of p-mTOR that were downregulated by Bz-ATP treatment. It also increased the expression level of *PERK*, *caspase-12*, and *CHOP*; however, this effect was reversed by GSK2606414, without affecting the levels of p-mTOR, LC3B, and Beclin-1. Flow cytometry results demonstrated that MHY1485 further increased the percentage of apoptotic chondrocytes upregulated by Bz-ATP (Figure 6D), along with the intracellular oxidative stress level (Figure 6E); however, GSK2606414 reversed this effect. Caspase-3 activity assay (Figure 6F) showed that apoptosis induced by MHY1485 was effectively inhibited by GSK2606414. These results suggest that mTOR promoted ER stress-apoptosis via PERK.

### Inositol-Requiring Enzyme-1 Inhibits mTOR, Promotes Autophagy, and Reduces ER Stress-Apoptosis

**FIGURE 7:**
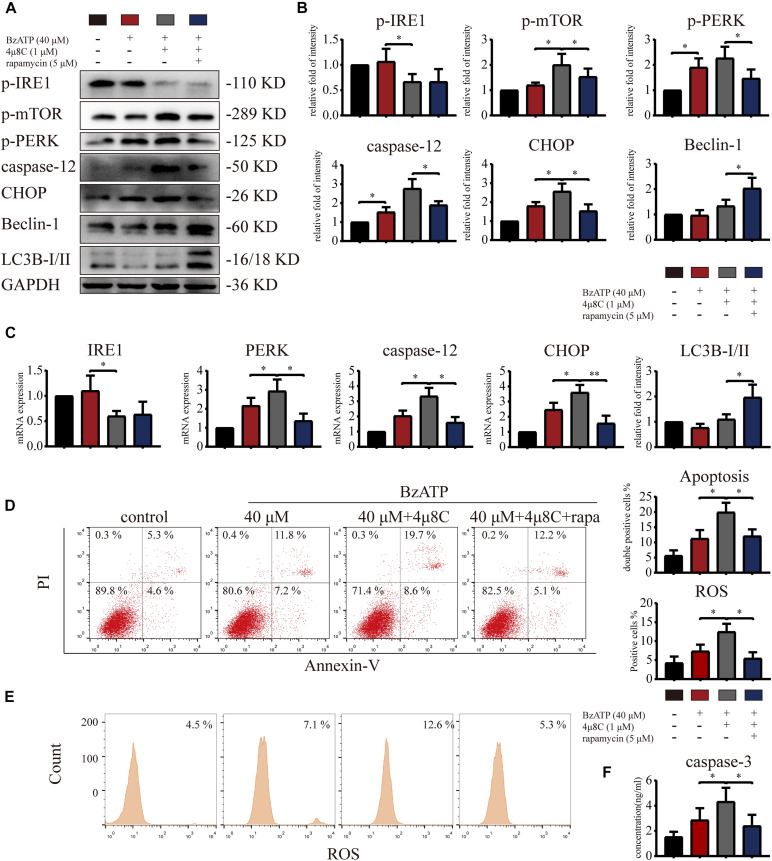
Inositol-requiring enzyme-1 inhibits mTOR and reduces ER stress-apoptosis. Cells were cultured with 40 μM Bz-ATP, 1 μM 4μ8C, and 5 μM rapamycin. Western blotting **(A,B)** and qRT-PCR **(C)** were used to detect the protein and mRNA expression levels of *IRE1*, mTOR, *PERK*, *caspase-12*, *CHOP*, LC3B, and Beclin-1. Flow cytometry was used to detect the percentage of double-stained Annexin-V/PI cells **(D)** and the level of intracellular oxidative stress, ROS **(E)**. **(F)** The caspase-3 activity assay; the statistical results are homogenized. Data are expressed as means ± standard deviations of at least three independent experiments. ^∗^*p* < 0.05, ^∗∗^*p* < 0.01.

To explore whether IRE1 is involved in the induction of apoptosis via the regulation of mTOR, we treated primary chondrocytes with 4μ8C (IRE1 inhibitor) and rapamycin (mTOR inhibitor) using the following experimental design: control, Bz-ATP (40 μM), Bz-ATP + 4μ8C (1 μM), and Bz-ATP + 4μ8C + rapamycin (5 μM). Western blotting (Figures 7A,B) and qRT-PCR (Figure 7C) results showed that 4μ8C inhibited the expression of IRE1, and increased mTOR phosphorylation. 4μ8C further increased the expression level of *PERK*, *caspase-12*, and *CHOP*, and this effect was reversed by rapamycin. Interestingly, rapamycin treatment did not have an effect on the expression levels of *IRE1*, as well as its phosphorylation levels. Flow cytometry experiments demonstrated that 4μ8C increased the percentage of apoptotic chondrocytes upregulated by Bz-ATP (Figure 7D) and the intracellular oxidative stress level (Figure 7E), while rapamycin treatment reversed this effect. The caspase-3 activity assay (Figure 7F) showed that 4μ8C-induced apoptosis was effectively inhibited by rapamycin. These results suggest that IRE1 inhibited mTOR to stimulate autophagic flux and reduce ER stress-apoptosis.

### Intra-Articular Injection of mTOR Inhibitor Rapamycin Promotes Chondrocyte Autophagy and Effectively Reduces OA Development

**FIGURE 8:**
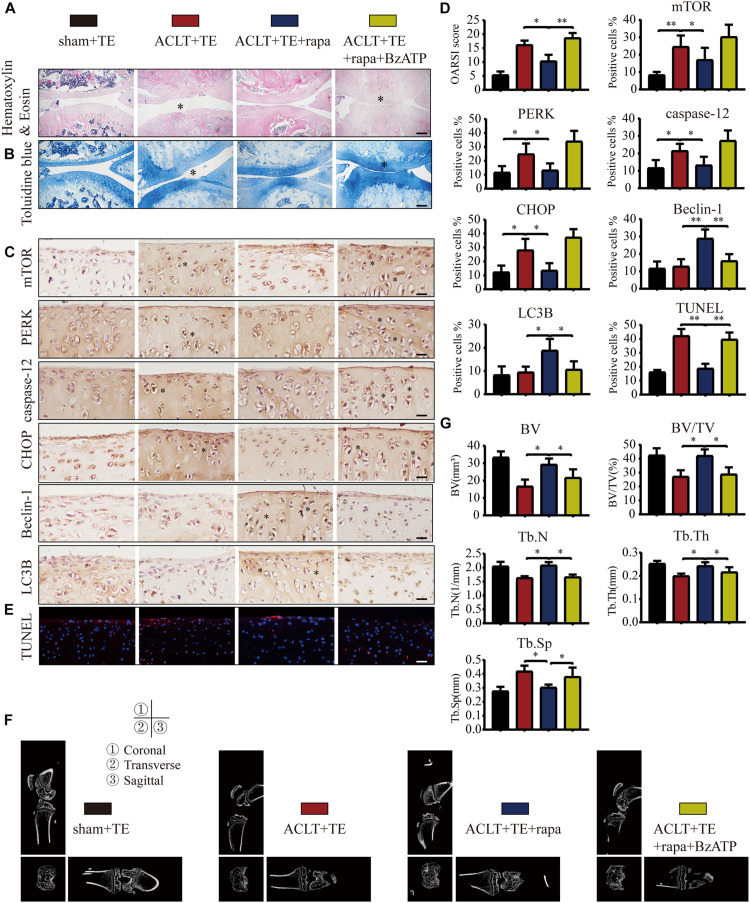
Inhibition of mTOR promotes autophagy, decreases apoptosis, and reduces cartilage loss. The sagittal sections of knee joints were stained with H&E **(A)** and TB **(B)** at the 8-week time point (scale bar: 500 μm). The degree of cartilage loss and matrix degradation was quantified using the OARSI scoring system; data are presented on the right. **(C,D)** IHC staining and quantification of mTOR, PERK, caspase-12, CHOP, LC3B, and Beclin-1 were performed to observe changes in the expression levels of these markers after intra-articular injections of rapamycin and Bz-ATP (scale bar: 200 μm). **(E)** TUNEL staining was performed to detect the number of apoptotic chondrocytes in each group (scale bar: 200 μm). **(F)** Micro-CT was used to evaluate the knee joints in each group and to obtain the imaging data of the tibial plateau and subchondral bone (scale = 1 mm). **(G)** Quantification of micro-CT bone-related parameters, including BV, BV/TV, Tb.N, Tb.Th, and Tb.Sp. The asterisk in the HE and TB staining images represents the cartilage damage site, and the asterisk in the histochemical images represents the positively stained cells. Data are expressed as means ± standard deviations of at least three independent experiments. ^∗^*p* < 0.05, ^∗∗^*p* < 0.01.

Our results so far (Figures 3–7) suggested that P2X7-mediated Ca^2+^ influx promoted apoptosis in chondrocytes through the IRE1-mTOR-PERK axis. Therefore, we tested whether mTOR inhibition could effectively alleviate OA symptoms using the following experimental design: sham + TE, ACLT + TE, ACLT + TE + rapamycin, and ACLT + TE + rapamycin + Bz-ATP and observed changes over 8 weeks. H&E and TB staining (Figures 8A,B) showed that the intra-articular injection of rapamycin (5 μM and 50 μL) significantly reduced the loss of knee articular cartilage and matrix degradation; however, this effect was reversed by injection of the P2X7 agonist Bz-ATP (40 μM and 50 μL). IHC analysis (Figures 8C,D) showed that rapamycin treatment downregulated the expression levels of mTOR, PERK, caspase-12, and CHOP and upregulated the expression levels of LC3B and Beclin-1; however, these effects were reversed by Bz-ATP treatment. TUNEL staining (Figure 8E) demonstrated that rapamycin reduced the percentage of apoptotic chondrocytes, whereas Bz-ATP increased the number of apoptotic cells. The micro-CT analysis of rat knee joints (Figures 8F,G) showed that bone destruction in the ACLT (OA) group was decreased by rapamycin injections; furthermore, osteophyte formation and joint space narrowing were also reduced. However, the injection of Bz-ATP reversed the anti-inflammatory effect of rapamycin, leading to more severe OA.

### Intra-Articular Injection of the mTOR Activator MHY1485 Inhibits Chondrocyte Autophagy and Accelerates OA Development

**FIGURE 9:**
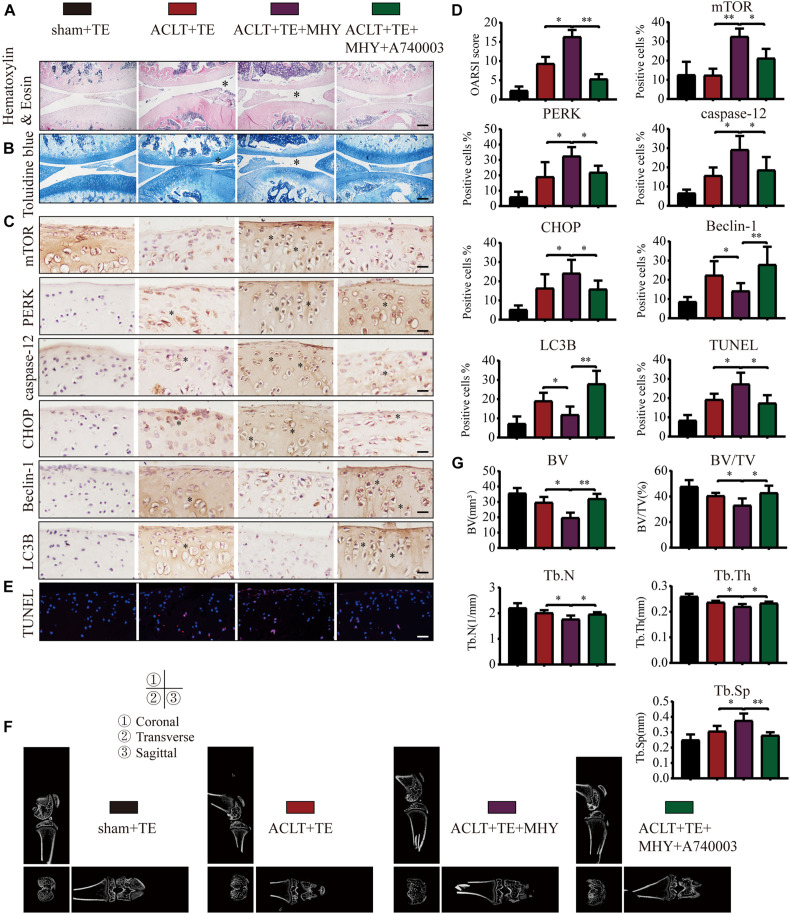
mTOR activation inhibits autophagy, induces apoptosis, and increases cartilage loss. The sagittal sections of knee joints were stained with H&E **(A)** and TB **(B)** at 4-week time point (scale bar: 500 μm). The degree of cartilage loss and matrix degradation was quantified using the OARSI scoring system; data are presented on the right. **(C,D)** IHC staining and quantification of mTOR, PERK, caspase-12, CHOP, LC3B, and Beclin-1 was performed to observe changes in the expression levels of these indicators after the intra-articular injections of rapamycin and Bz-ATP (scale bar: 200 μm). **(E)** TUNEL staining was performed to quantify the number of apoptotic chondrocytes in each group (scale bar: 200 μm). **(F)** The knee joints in each group were evaluated using micro-CT, and the imaging data of the tibial plateau and subchondral bone were obtained (scale = 1 mm). **(G)** Quantification of micro-CT bone-related parameters, including BV, BV/TV, Tb.N, Tb.Th, and Tb.Sp. The asterisk in the HE and TB staining images represents the cartilage damage site, and the asterisk in the histochemical images represents the positively stained cells. Data are expressed as means ± standard deviations of at least three independent experiments. ^∗^*p* < 0.05, ^∗∗^*p* < 0.01.

To further characterize the role of mTOR in OA, rats were injected with mTOR activator MHY1485 using the following experimental design: sham + TE, ACLT + TE, ACLT + TE + MHY1485, and ACLT + TE + MHY1485 + A740003 at 4 weeks. H&E and TB staining (Figures 9A,B) showed that an intra-articular injection of MHY1485 (10 μM and 50 μL) significantly accelerated the loss of knee articular cartilage and matrix degradation, while this effect was reversed by an injection of P2X7 inhibitor A740003 (20 μM and 50 μL). IHC analysis results (Figures 9C,D) demonstrated that MHY1485 upregulated the expression levels of mTOR, PERK, caspase-12, and CHOP and downregulated the expression levels of LC3B and Beclin-1; however, these effects were reversed by A740003. TUNEL staining (Figure 9E) also confirmed that MHY1485 increased the percentage of apoptotic chondrocytes, while A740003 reduced the number of apoptotic cells. The micro-CT analysis of rat knee joints (Figures 9F,G) showed that bone destruction in the ACLT group was increased by MHY1485, and enhanced osteophyte formation and joint space narrowing were observed. The injection of A740003 appeared to enhance the anti-inflammatory effect of exercise to reduce OA.

## Discussion

The prevention and treatment of OA are among the most urgent healthcare problems in today’s society (Hunter and Bierma-Zeinstra, 2019). Exercise is considered to be a simple and economical way to prevent OA. However, moderate exercise increases joint stability, whereas excessive exercise aggravates joint damage. P2X7 is a key molecule involved in the regulation of inflammation, as well as other numerous cellular responses dependent on the type and level of stimulation. For example, over-activation of P2X7 induces damage or apoptosis (Li et al., 2021a). We observed that moderate-intensity exercise did not affect the expression levels of P2X7 in healthy rats, while the expression level of P2X7 gradually increased with time in the ACLT-induced OA rats. During the pre-exercise period (2–4 weeks), P2X7 was moderately activated and mediated IRE1 expression and the level of autophagy. During the late exercise period (6–8 weeks), P2X7 was over-activated, IRE1 levels and autophagy were decreased, and cartilage loss and matrix degradation were significantly accelerated. During the entire exercise process (2–8 weeks), PERK and PERK-mediated apoptosis signals were activated. Furthermore, our results demonstrated that mTOR played a role by suppressing autophagy and promoting ER stress-apoptosis.

Numerous factors have been shown to induce and moderate ER stress to maintain cellular homeostasis. Similar to extracellular ATP (Chao et al., 2012), we found that Bz-ATP activated P2X7-mediated Ca^2+^ influx resulting in cytoplasmic Ca^2+^ overload, interfering with the ER Ca^2+^ homeostasis, inducing ER stress, and ultimately leading to cell death. Downstream of mTOR, PERK activation prevented autophagosome-lysosome fusion in response to chronic ER stress, and mediated ER stress-apoptosis. It has been previously shown that lysosomal dysfunction leads to the accumulation of incompletely degraded glycosaminoglycans, affecting the structure of the extracellular matrix (ECM). Furthermore, insufficient or increased secretion of dysfunctional ECM proteins occurs in the ER, resulting in ER stress, resulting in changes in the cartilage (Madhu et al., 2020). mTOR is a negative regulator of autophagy. The inhibition of mTOR, either using selective inhibitors (Ito et al., 2017) or cartilage-specific gene knockouts (Vasheghani et al., 2015), decreases senescence, chondrocyte apoptosis, and ECM degradation and reduces meniscal instability and other symptoms of OA. Being upstream to mTOR, the IRE1 gene is knocked down to accelerate ER stress-apoptosis (Han et al., 2013).

**FIGURE 10:**
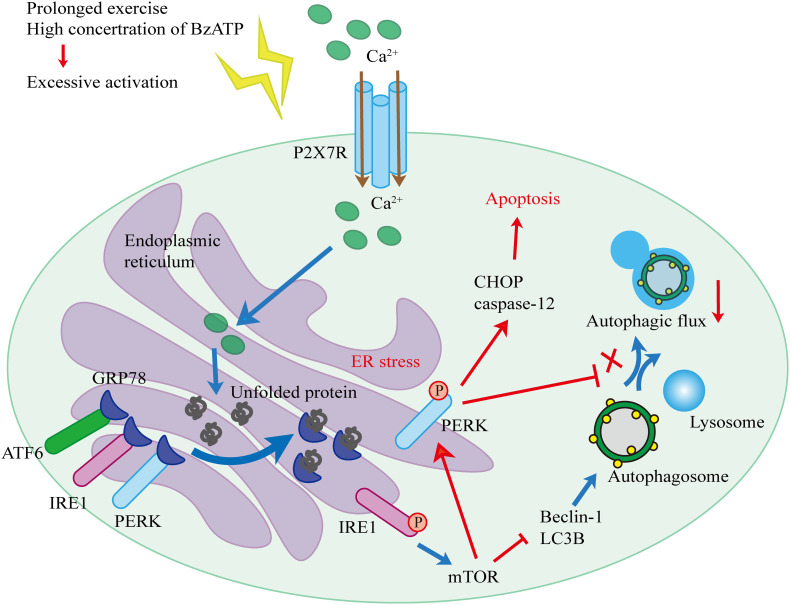
Schematic diagram of the working hypothesis.

Based on our findings and published literature, we propose the following hypothesis (Figure 10). First, biomechanical forces generated by moderate-intensity exercise activated P2X7, leading to the Ca^2+^ influx and Ca^2+^ overload in the ER, and resulting in the induction of ER stress. Our results showed that the levels of GRP78, a Ca^2+^-dependent molecular chaperone and one of the markers of ER stress (Cerezo and Rocchi, 2017), increased in response to exercise. ER stress in chondrocytes initiated the UPR and upregulated the expression levels of PERK, IRE1, and ATF6 to maintain ER homeostasis. During prolonged exercise (or high Bz-ATP concentrations), the activation of P2X7 gradually increased, and UPR was not able to compensate for the continuous ER stress. As a result, both IRE1-mediated autophagic flux and PERK-mediated ER stress-apoptosis were activated. In the later stage of the experiment, the expression levels of IRE1 began to decline. mTOR did not block the autophagic flux, while its downstream target, PERK, prevented the fusion of autophagosomes and lysosome. At the same time, the inhibition of PERK and mTOR restored the autophagic flux and effectively reduced ER stress-apoptosis. Inhibition of IRE1 also showed that the decrease in its expression level leads to the loss of mTOR inhibition, such that mTOR converts autophagy into apoptosis. Furthermore, intra-articular injection of MHY1485 and rapamycin confirmed that the IRE1-mTOR-PERK axis was involved in the regulation of autophagy and apoptosis in chondrocytes.

Here, we analyzed the role of P2X7 (and its downstream pathways) in exercise as a therapy for OA. We believe that the level of mechanical stress stimulation may determine the role of P2X7. In addition to its role in chondrocytes, in response to high mechanical stress, P2X7 and ATP signaling are also involved in the formation of osteoclasts (Bratengeier et al., 2019), while P2X7 inhibitors can effectively suppress osteoclastogenesis and maintain bone homeostasis (Pellegatti et al., 2011). Furthermore, in the case of micro-injury, P2X7 acts as a repair receptor to maintain bone remodeling and health (Jørgensen, 2018), by regulating the balance between bone resorption and bone formation and by inducing bone formation in response to mechanical load to relieve stress fracture injury (Zeng et al., 2019). In addition, oxidative stress, autophagy, and apoptosis could also be targets of the treatment. For example, mitochondrial dysfunction caused by ROS and mtDNA damage disrupts the balance and stability of chondrocyte anabolism. In response to environmental pressures, the chondrocyte metabolic pathway changes, and the ATP produced by compensation promotes the synthesis of pro-inflammatory and degradable proteins (Zheng et al., 2021), suggesting that P2X7 may play an important role in this pathway as well. The autophagic degradation of organelles, such as mitochondrial autophagy (Quinsay et al., 2010) and ER autophagy (Nakatogawa and Mochida, 2015), can maintain the homeostasis of mitochondria and the ER and help reduce the occurrence of apoptosis. For example, icariin inhibits ER stress-mediated autophagy through the MAPK signaling pathway and decreases the apoptosis of bone marrow-derived mesenchymal stem cells caused by oxygen, glucose, and serum deprivation (Liu et al., 2020). Another natural compound, curcumin, alleviates cartilage degeneration and chondrocyte apoptosis in ACLT rats via inhibition of the PERK-eIF2α-CHOP signaling axis (Feng et al., 2019).

Here, we demonstrated that moderate-intensity exercise and duration, corresponding to the physiological levels of P2X7 activation, play a role in the prevention and alleviation of OA. However, as OA progresses to an advanced stage and becomes severe, treatments targeting P2X7 and its downstream pathways could provide an effective way to treat OA. Our work has several limitations. For example, for the functional P2X7 experiments, mechanical stretching (Beckel et al., 2014) and fluid shear (Zhang et al., 2017) *in vitro* models could be more representative of the *in vivo* conditions. These *in vitro* models will be used in our future experiments to further investigate the mechanism of P2X7 action in response to biomechanical forces.

## Conclusion

This is the first report proposing that in the OA model that includes exercise, P2X7 activation mediates Ca^2+^ influx, resulting in the regulation of autophagy and apoptosis through the IRE1-mTOR-PERK axis in chondrocytes. The autophagic flux mediated by IRE1 was inhibited by mTOR, as well as by PERK blocking the fusion of autophagosomes and lysosomes during the later time points. Furthermore, mTOR activated PERK to increase the expression of caspase-12 and CHOP and mediate ER stress-apoptosis. Therefore, mTOR, together with P2X7, provides a potential target for the treatment of OA.

## Data Availability Statement

All original data are available from the corresponding author upon request. Source data for Figures 3–7 is provided in the article.

## Ethics Statement

The animal study was reviewed and approved by the Animal Ethics Regulations of China Medical University (no. 2017PS237K).

## Author Contributions

ZL: conceptualization, methodology, and writing – original draft. ZL, HZ, JL, YW, and YY: formal analysis and resources. ZL, ZH, and LB: writing – review and editing. ZL and LB: funding acquisition and supervision. All authors contributed to the article and approved the submitted version.

## Conflict of Interest

The authors declare that the research was conducted in the absence of any commercial or financial relationships that could be construed as a potential conflict of interest.
