# Supplementary material for: IRE1-mTOR-PERK Axis Coordinates Autophagy and ER Stress-Apoptosis Induced by P2X7-Mediated Ca2+ Influx in Osteoarthritis
Source: Front Cell Dev Biol. 2021 Jun 17;9:695041. doi: 10.3389/fcell.2021.695041 (PMC8248364; doi:10.3389/fcell.2021.695041)
Supplement: Supplementary file 1 [file Data_Sheet_1.docx]

Supplementary Material


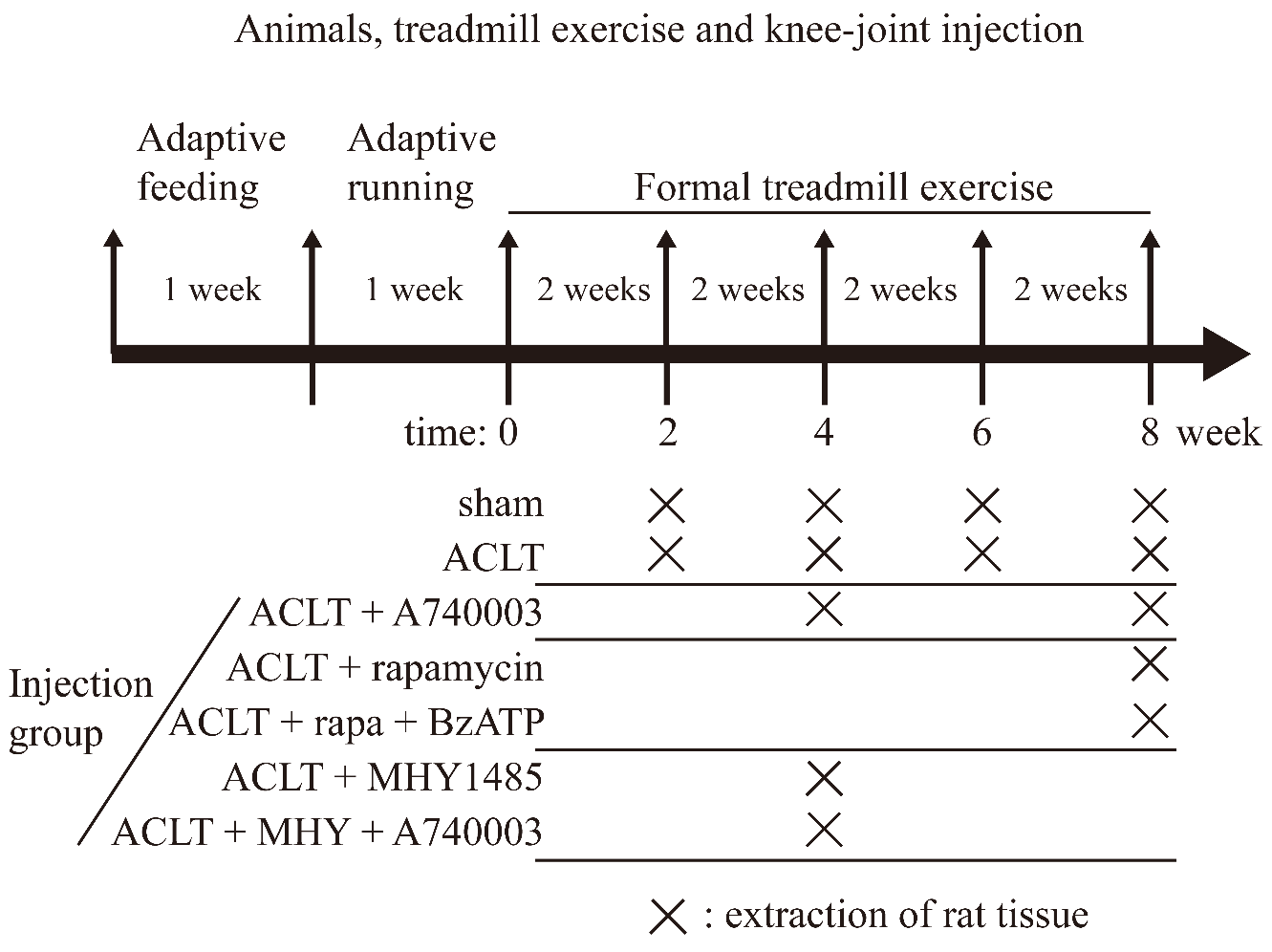


# Figure S1. Schematic overview of experimental design. Detailed experimental arrangements for “Animals, treadmill exercise, and knee-joint injections” in the Materials and Methods section.

# Figure S2: Rat primer sequences

| Gene Name | Species |  | Sequence |
| --- | --- | --- | --- |
| P2X7 | rat | forward | 5′- TTAGTACACGGCATCTTCGACACG - 3′ |
|  |  | reverse | 5′- AGCTTCTGTTCTTGGCCTTCTGAC - 3′ |
| GRP78 | rat | forward | 5′- TCAGCCCACCGTAACAATCAAG - 3′ |
|  |  | reverse | 5′- TCCAGTCAGATCAAATGTACCCAGA - 3′ |
| PERK | rat | forward | 5′- CCAAGCTGTACATGAGCCCAGA -3′ |
|  |  | reverse | 5′- TTTCTGAGTGAACAGTGGTGGAAAC -3′ |
| IRE1 | rat | forward | 5′- CATCACCATGTATGACACCAAGACC - 3′ |
|  |  | reverse | 5′- TGTCCACAGTTACCACCAGTCCA - 3′ |
| ATF6 | rat | forward | 5′- ATCACCTGCTATTACCAGCTACCAC - 3′ |
|  |  | reverse | 5′- TGACCTGACAGTCAATCTGCATC - 3′ |
| CHOP | rat | forward | 5′- TGGAAGCCTGGTATGAGGATCTG - 3′ |
|  |  | reverse | 5′- GAGGTGCTTGTGACCTCTGCTG - 3′ |

| caspase-12 | rat | forward | 5′- CAATTCCGACAAACAGCTGAGTTTA - 3 ′ |
| --- | --- | --- | --- |
|  |  | reverse | 5 ′- CATGGGCCACTCCAACATTTAC - 3′ |
| GAPDH | rat | forward | 5′- GGCACAGTCAAGGCTGAGAATG - 3′ |
|  |  | reverse | 5′- ATGGTGGTGAAGACGCCAGTA - 3′ |


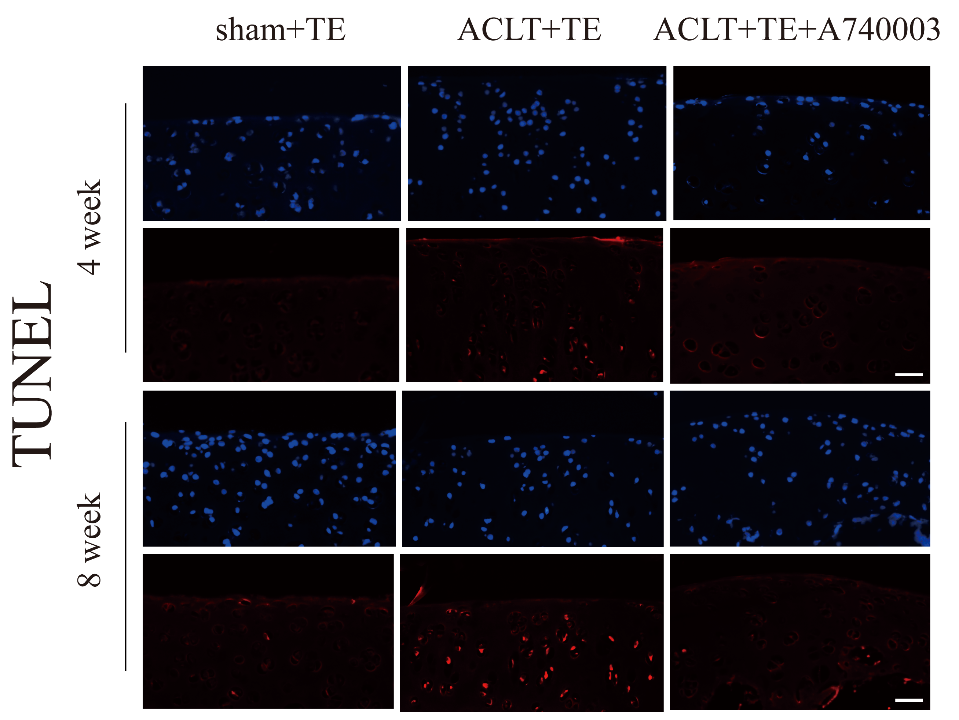


# Figure S3. TUNEL staining of rat knee joints. Separated fluorescent channels corresponding to the merged images presented in Figure 2F (scale bar: 200 μm).


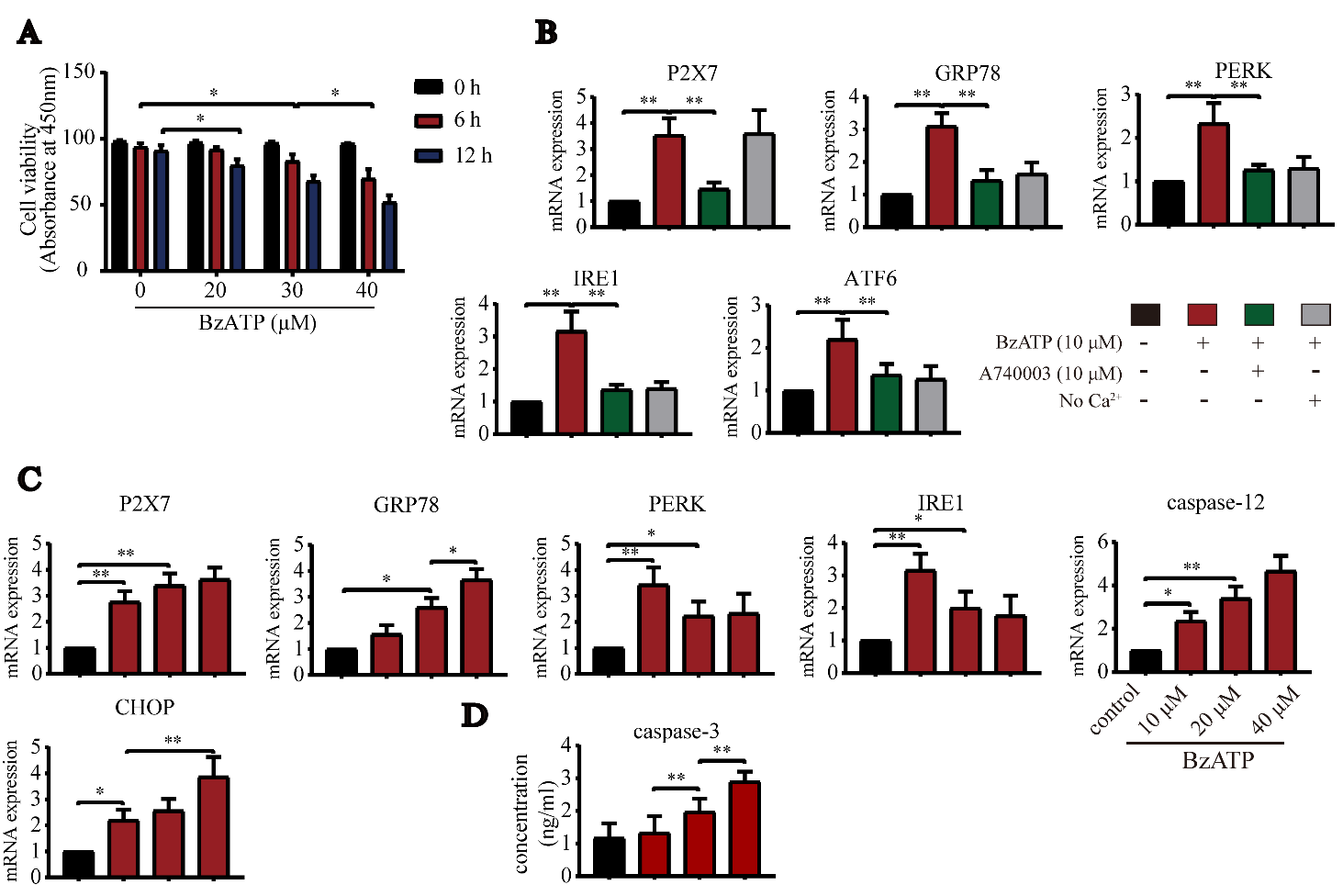


**Figure S4**. **Supplement to Figure 3.** (A) CCK-8 assay. Cells were cultured with different Bz-ATP concentrations (0, 10, 20, and 40 μM) for 6 and 12 h, and cell viability was evaluated. (B) qRT-PCR. Cells were cultured with 10 μM Bz-ATP, P2X7 selective inhibitor A740003, or Ca^2+^-free medium as indicated. The mRNA expression levels of *GRP78*, *PERK*, *IRE1*, and *ATF6* were evaluated. (C) qRT-PCR. Cells were cultured with 10 μM Bz-ATP, and the expression levels of *P2X7*, *GRP78*, *caspase-12*, and *CHOP* were evaluated by qRT-PCR. (D) Caspase-3 activity assay. Cells were cultured with 10 μM Bz-ATP, and caspase-3 activity was evaluated as described in the Materials and Methods section. Data are expressed as means ± standard deviations of at least three independent experiments. ^⁎^*p* < 0.05, ^⁎⁎^*p* < 0.01.


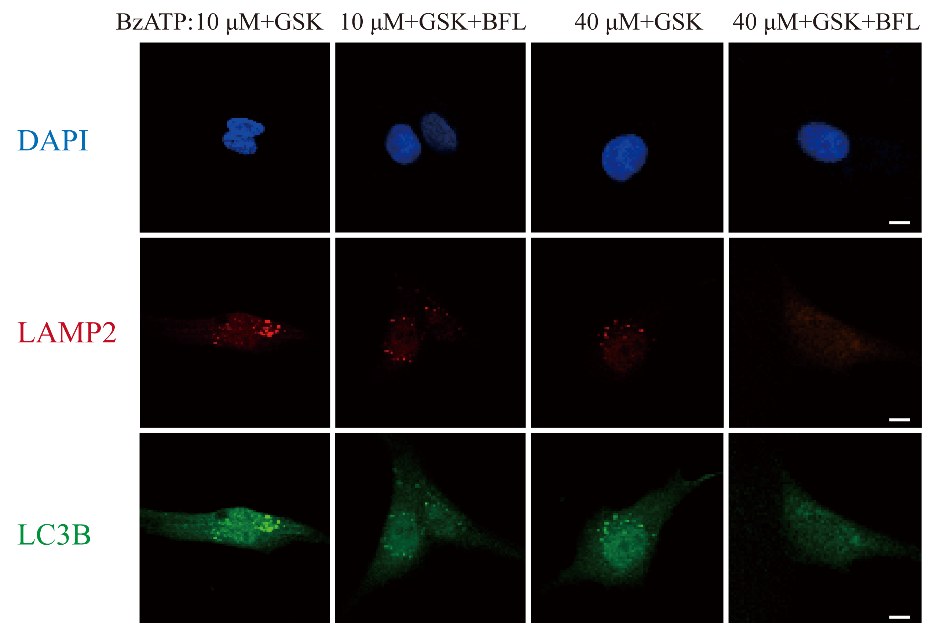


# Figure S5. Immunofluorescence staining of primary chondrocytes. Separated fluorescent channels corresponding to the merged images presented in Figure 5G (LC3B: green, LAMP2: red, DAPI: blue) (scale bar: 5 μm).


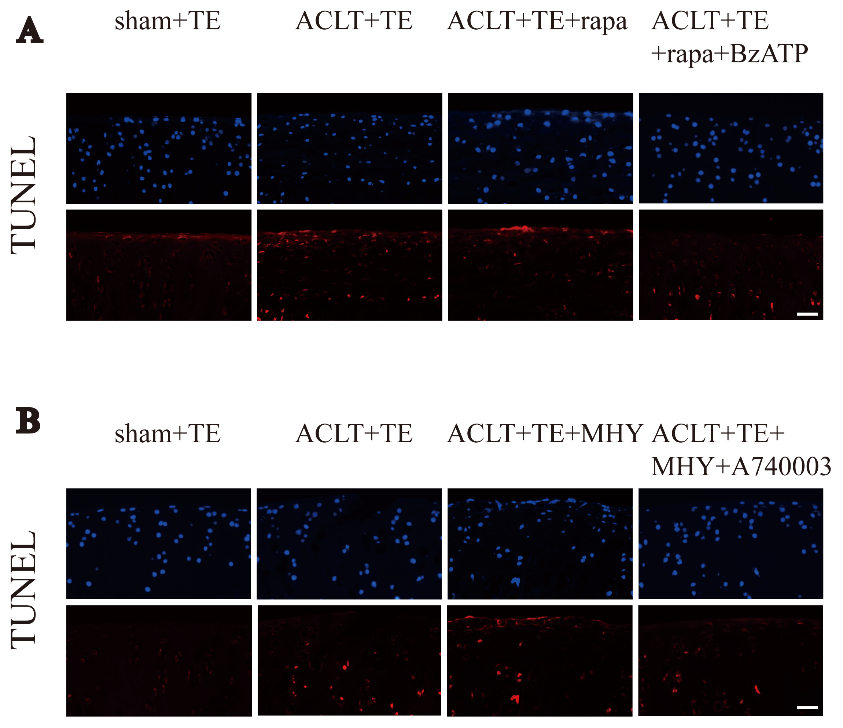


# Figure S6. TUNEL staining of rat knee joints. Separated fluorescent channels corresponding to the merged images presented in Figures 8E (A), and 9E (B) (scale bar: 200 μm).
